# The Role of Oxidative Stress and Natural Antioxidants in Ovarian Aging

**DOI:** 10.3389/fphar.2020.617843

**Published:** 2021-01-14

**Authors:** Liuqing Yang, Yun Chen, Yan Liu, Yu Xing, Chenyun Miao, Ying Zhao, Xiangwei Chang, Qin Zhang

**Affiliations:** ^1^ Guangxing Hospital Affiliated to Zhejiang Chinese Medical University, Hangzhou, China; ^2^ The 2nd Clinical Medical College, Zhejiang Chinese Medical University, Hangzhou, China; ^3^ Brigham and Women’s Hospital and Harvard Medical School, Boston, MA, United States; ^4^ Dongfang Hospital, Beijing University of Chinese Medicine, Beijing, China; ^5^ College of Pharmacy, Anhui University of Chinese Medicine, Hefei, China

**Keywords:** oxidative stress, natural antioxidants, anti-ovarian aging, reactive oxygen species, mitochondria, inflammation, apoptosis, telomeres

## Abstract

The ovarian system comprises vital organs in females and is of great significance for the maintenance of reproductive potential and endocrine stability. Although complex pathogenesis undoubtedly contributes to ovarian aging, increasing attention is being paid to the extensive influence of oxidative stress. However, the role of oxidative stress in ovarian aging is yet to be fully elucidated. Exploring oxidative stress-related processes might be a promising strategy against ovarian aging. In this review, compelling evidence is shown that oxidative stress plays a role in the etiology of ovarian aging and promotes the development of other ovarian aging-related etiologies, including telomere shortening, mitochondrial dysfunction, apoptosis, and inflammation. In addition, some natural antioxidants such as quercetin, resveratrol, and curcumin have a protective role in the ovaries through multiple mechanisms. These findings raise the prospect of oxidative stress modulator-natural antioxidants as therapeutic interventions for delaying ovarian aging.

## Introduction

Dreams of longevity are as old as humanity itself. Over the past decades, human longevity has increased substantially worldwide. It is predicted that approximately 22% of the global population will be older than 60 by 2050 (Bellantuono, 2018). Consequently, age-related diseases, such as cognitive impairment, cardiovascular diseases, and cancer are markedly increasing and are the leading cause of death and reduced quality of life (Franceschi et al., 2018a).

Ovarian state influence the health of all phases of life and are the main determinants of female life span (de Kat et al., 2016; Rajkovic and Pangas, 2017; Mason et al., 2018; Quinn and Cedars, 2018; Tsiligiannis et al., 2019). Ovarian aging, which occurs earlier than the aging of most other organs, is a continuous process starting from the oocyte death of the embryo at 20 weeks of gestation. (Amanvermez and Tosun, 2016). The most significant characteristic of ovarian aging is a diminished ovarian reserve (DOR), i.e., the decline in the quality and quantity of oocytes, which is also the main reason for infertility and failure in assisted reproductive technology (ART) (May-Panloup et al., 2016). Moreover, ovarian aging is associated with an increased risk for diabetes, heart disease, cancer, and other age-related conditions. The importance of the ovary in maintaining health and extending lifespan has also been well demonstrated in animal models. Transplanting the ovaries of young mice into old mice can prolong the life span of the old mice (Mason et al., 2018). Cardioprotective benefits, cognitive behavior, and immune and renal functions can be positively restored by re-establishment of active ovarian function in aged female mice (Mason et al., 2011; Parkinson et al., 2017; Peterson et al., 2017). Therefore, keeping the ovaries “young” is critical.

Several theories have been proposed to explain the mechanism underlying ovarian aging, including free radical theory, apoptosis, telomere shortening, mitochondrial dysfunction, and inflammation theory (Shi et al., 2016; Regan et al., 2018a; Huang Y et al., 2019; Kasapoğlu and Seli, 2020; Wang et al., 2020). Free radical theory, a classical theory of aging, proposes that oxidative stress caused by elevated intracellular levels of reactive oxygen species (ROS) is the most significant contributor to cellular senescence and aging in mammals (Liochev, 2013; Zhang H et al., 2015). Furthermore, numerous studies have documented that oxidative stress is a leading driver of the ovarian aging process and promotes the development of other ovarian aging-related etiologies, such as telomere shortening, mitochondrial dysfunction, apoptosis, and inflammation (Lim and Luderer, 2011; Arulselvan et al., 2016; Bauer et al., 2017; Prasad et al., 2017; Takeo et al., 2017; Huang ML et al., 2019; Sasaki et al., 2019; Pineda-Pampliega et al., 2020). The role of oxidative stress is also becoming increasingly evident in the pathogenesis of a diverse range of pathological conditions, including Alzheimer’s disease, cardiometabolic diseases, cancer, diabetes mellitus, and retinal dystrophies (Niemann et al., 2017; Saha et al., 2017; Butterfield and Boyd-Kimball, 2018; Cioffi et al., 2019; Domènech and Marfany, 2020; Yaribeygi et al., 2020).

Based on these theoretical considerations, alleviating oxidative stress in the ovaries is an important means of delaying ovarian aging. Effective natural antioxidants could provide novel and safe interventional strategies to delay or prevent ovarian aging and related diseases. Despite a series of reports regarding the antioxidative effects of natural products on ovarian aging, to date, there are no related systematic reviews in the literature. In this review, the underlying mechanism of oxidative stress during ovarian aging and the molecular protective mechanisms of natural antioxidants in anti-ovarian aging are comprehensively explored.

## Overview of Oxidative Stress in Ovarian Aging

ROS, including both free radical and non-free radical, oxygenated molecules, such as superoxide radicals (O_2_•−), H_2_O_2_, hydroxyl radicals (•OH), and singlet oxygen (^1^O_2_), are mostly generated continuously as byproducts during common metabolic processes in eukaryotic cells (Liguori et al., 2018). They all contain oxygen atoms and have strong oxidizing abilities. A few reviews summarized the pathophysiological functions of ROS (Sugino, 2005; Dizdaroglu and Jaruga, 2012; Rizzo et al., 2012; Kamat et al., 2016; Lu et al., 2018). At low levels, ROS widely participate in cell signal transduction and promote cell survival, proliferation, and differentiation (Rizzo et al., 2012; Lu et al., 2018). ROS are also physiological regulators of ovarian processes and play a key role in follicular development and survival (Sugino, 2005). However, heightened levels of ROS, which overpower cellular antioxidant and repair capacities, can trigger oxidative stress in cells, directly causing oxidative damage to all biomolecules within the cellular environment (including proteins, lipids, and DNA), and thus contribute to the development of aging and related diseases (Dizdaroglu and Jaruga, 2012; Kamat et al., 2016).

Oxidative stress, caused by the imbalance between the production and destruction of ROS, directly damages the intraovarian environment, just as it does in many other cells. Moreover, all primary oocytes are formed by the fifth month of fetal life and remain dormant before complete meiosis I, a decades-long process rendering the oocyte susceptible to chronic oxidative insult (Peters et al., 2020). To date, several studies have shown that the accumulation of ROS in the ovaries deteriorates oocyte quality, induces granulosa cell (GC) apoptosis, and accelerates degeneration of the corpus luteum (Chaube et al., 2014; Tiwari et al., 2015; Prasad et al., 2016; Wang S. et al., 2017; Yang et al., 2017; Sohel et al., 2019). Furthermore, it decreases communication between oocytes and GCs, affecting preovulatory oocyte maturation (Cajas et al., 2020). Oxidative damage to the ovary is generally caused by the propagation of lipid peroxidation cascades, which seriously influences folliculogenesis, meiosis, and ovulation, and eventually leads to ovarian aging (Lim and Luderer, 2011; Edrey and Salmon, 2014; Maclaran and Nikolaou, 2019).

The level of intra-ovarian ROS has been confirmed to be positively correlated with female age, which also makes the female germline particularly vulnerable to the cumulative effects of chronic oxidative stress (Lim and Luderer, 2011; Takeo et al., 2017; Sasaki et al., 2019). Age-related increases in ROS have been found in the follicular fluid and oocytes of women undergoing ART. Among these elderly women with high ROS levels, the success rate of ART is relatively low (Oyawoye et al., 2003; Wiener-Megnazi et al., 2004). In contrast, the content of antioxidants in the intraovarian environment is reduced with age, which means there is a diminished ability to scavenge ROS. Superoxide dismutases (SODs) and catalase (CAT) are the major endogenous antioxidant defense systems against ROS (Racchi, 2013; Ighodaro and Akinloye, 2018). Clinical studies have shown that the mRNA and protein levels of the aforementioned antioxidants in GCs of elderly women undergoing *in vitro* fertilization (IVF) were significantly lower than those of younger women. The elderly group also showed abnormal mitochondrial morphology and fewer lipid droplets at the ultrastructural level (Tatone et al., 2006). In addition, Matos et al. presented evidence that SOD activity and SOD1 levels in cumulus cells surrounding ovulated oocytes decreased remarkably with female age, and low SOD activity is related to unsuccessful IVF outcomes (Matos et al., 2009). In conclusion, the imbalance between ROS and antioxidants leads to a decline in oocyte quality, which is an important factor affecting the success of ART and is implicated in the aging process of the ovaries.

In addition to direct damage to the ovaries, oxidative stress can also promote the development of other ovarian aging-related mechanisms, including telomere shortening, mitochondrial dysfunction, inflammation, and apoptosis.

## Effects of Oxidative Stress on Telomeres in the Ovaries

### Telomeres and Ovarian Aging

Telomeres, the dynamic nucleoprotein–DNA structures present at both ends of each chromosome, are responsible for maintaining genome integrity and chromosome stability (Zhang et al., 2018). The length of telomeres shortens progressively with each cell division (mitosis) and is strongly associated with lifespan (Heidinger et al., 2012; Turner et al., 2019). Excessive shortening triggers persistent DNA damage response or genomic instability, causing cellular senescence, and is also strongly linked to lifespan (Whittemore et al., 2019; Smith et al., 2020). Recently, the correlation between the telomere status of ovarian cells and the human female reproductive lifespan has drawn growing attention. A cross-sectional study indicated that the lack of human GC telomerase activity was associated with occult ovarian insufficiency (Butts et al., 2009). Additionally, shortened telomere length and diminished telomerase activity in GCs have also been associated with primary ovarian insufficiency (POI), thereby serving as potential molecular markers for the progression of ovarian function decline (Xu X et al., 2017). Telomere length in cumulus cells is positively correlated with oocyte and embryo quality (Cheng et al., 2013). Consistent with this, Keefe et al. found that in IVF, the pregnancy rate is higher in women with longer oocyte telomere length than in those with shorter oocyte telomere lengths (Keefe et al., 2007).

### Oxidative Stress and Telomeres in the Ovaries

**FIGURE 1:**
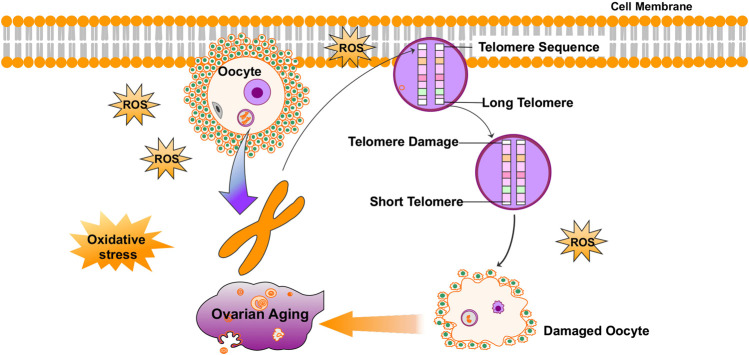
Oxidative stress mediates telomere damage in the ovaries and causes ovarian aging.

A few reviews summarized that telomeres intrinsically comprise hundreds of guanine bases, which is most easily oxidized by ROS because they have the lowest redox potential amongst the DNA bases (Tardat and Déjardin, 2018). Furthermore, telomere oxidative lesions are less efficiently repaired because of the telomere heterochromatin state, suggesting that telomeres are most vulnerable to oxidative damage (Smith, 2018; Coluzzi et al., 2019; Singh et al., 2019). Accordingly, oxidative stress is considered to be the main cause of telomere shortening (Erusalimsky, 2020). Cigarette smoke, a source of exogenous pro-oxidants, has been confirmed to be associated with increased oxidative stress in the ovary (Sobinoff et al., 2013; Mai et al., 2014; Kim et al., 2018; Li et al., 2020). Related studies have shown that mice exposed to chronic cigarette smoke condensate or smoke exhibited increased egg fragmentation, delayed fertilization, increased apoptosis in blastocysts, and shortened telomeres in embryos (Huang et al., 2009; Dechanet et al., 2011). Furthermore, another study found that the smoke component-induced oxidative damage is greatly reduced by the antioxidant, N-acetylcysteine (NAC) (Huang et al., 2010). It has also been confirmed that increased ROS levels in oocytes result in telomere shortening and reduced developmental competence of aged oocytes (Sasaki et al., 2019). ROS levels are lower, and telomeres are longer, in oocytes from young females (6–8 weeks of age) than in those from reproductively aged female mice (42–48 weeks of age) (Yamada-Fukunaga et al., 2013). In summary, as an important factor of aging, oxidative stress can also mediate telomere damage in the ovaries and cause ovarian aging (Figure 1).

## Effects of Oxidative Stress on Mitochondria in the Ovaries

### Mitochondria and Ovarian Aging

Growing evidence suggests that there is a close relationship between mitochondria and ovarian aging (May-Panloup et al., 2016; Wang T et al., 2017; Kasapoğlu and Seli, 2020). Mitochondria act as energy factories for cells, providing energy and regulating cellular signaling pathways for oocyte maturation, fertilization, and embryogenesis through aerobic respiration (Zhang et al., 2017; Harvey, 2019). Studies have shown that blocking adenosine triphosphate (ATP) synthase prevents germ stem cells from developing into oocytes (Teixeira et al., 2015). Unlike Mendelian inheritance, mitochondrial DNA (mtDNA) is derived exclusively from maternal egg cells (Lutz-Bonengel and Parson, 2019). The mtDNA copy number of oocytes, GCs, and cumulus cells are being explored as predictors of oocyte quality and embryo viability (Wells, 2017; Cecchino and Garcia-Velasco, 2019). Ovarian aging is related to reduced oocyte mtDNA content, and studies have shown that the mtDNA copy number per oocyte of young women is significantly higher than that of aged women or those of the same age with DOR (Pasquariello et al., 2019). In addition, the accumulation of female mouse germline mtDNA mutations exacerbates ovarian aging and reduces lifespan (Ross et al., 2014; Yang et al., 2020). Evidence for this is that the mitochondrial content of GCs and oocytes was significantly decreased in a mouse model of fragile X primary ovarian insufficiency (Conca Dioguardi et al., 2016). Therefore, reproductive experts proposed that mitochondrial transplantation might be a novel possibility for rejuvenating oocyte quality and overcoming age-related infertility or recurrent IVF failure (Kristensen et al., 2017; Labarta et al., 2019). In humans as well as in other animal species, the transfer of autologous or heterologous mitochondria has been proven to improve oocyte quality and IVF outcomes (Hua et al., 2007; Oktay et al., 2015; Srirattana and St John, 2018). Therefore, mitochondria have multiple effects on ovarian aging.

### Oxidative Stress and Mitochondria in the Ovaries

**FIGURE 2:**
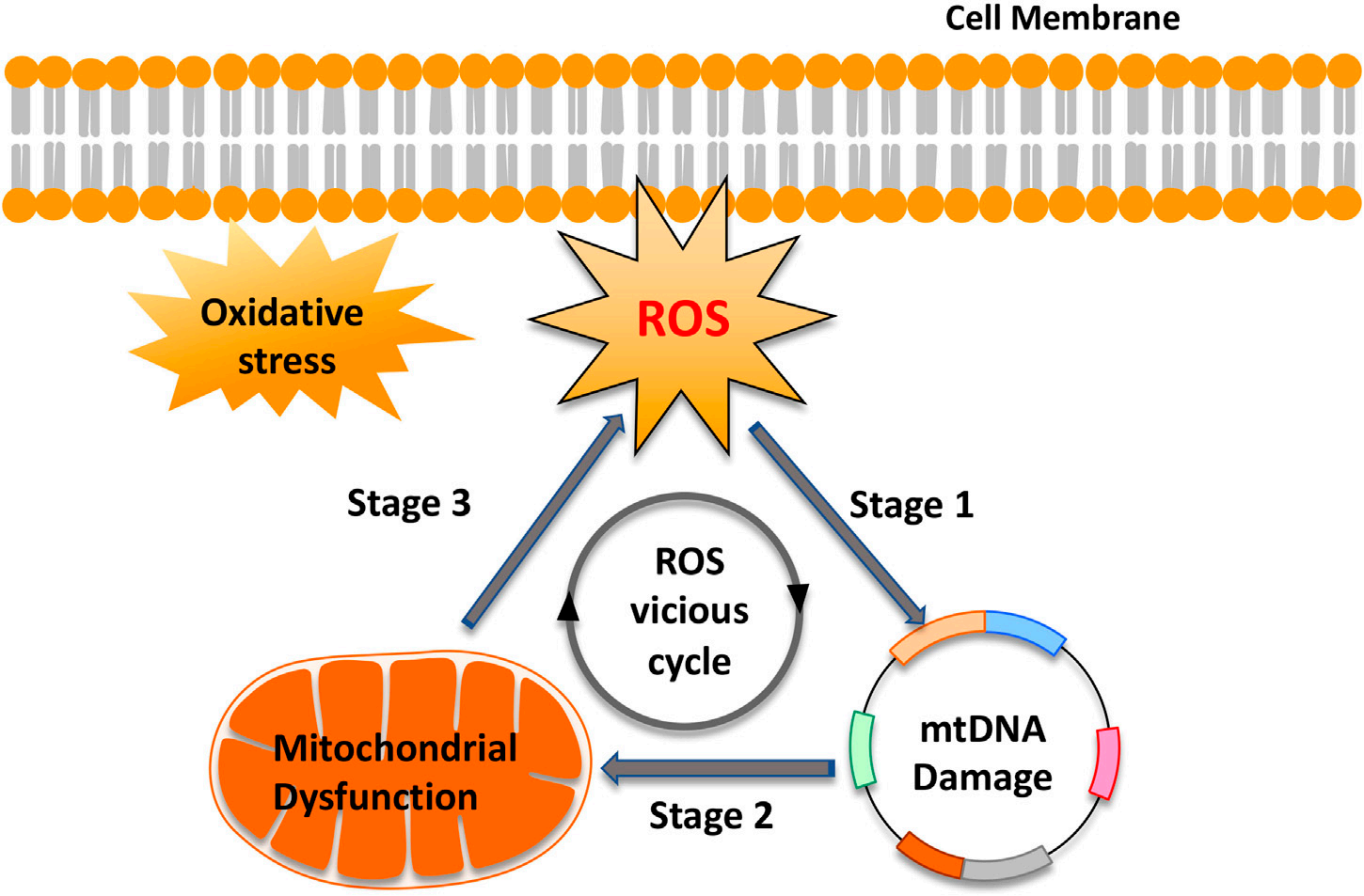
“Reactive oxygen species (ROS) vicious cycle” of ROS production, mtDNA damage, mitochondrial dysfunction, and further ROS production. The cycle implies an exponential growth of ROS production and mtDNA mutagenesis.

Mitochondria produce energy to drive the cell’s biochemical reactions. In addition, they are also the main source of ROS based on electron leakage from the respiratory chain (Scialò et al., 2017). However, paradoxically, mtDNA, because of the lack of protection by histones or DNA-binding proteins, is particularly vulnerable to ROS-mediated damage (Aryaman et al., 2017; Kauppila et al., 2017; Kaarniranta et al., 2019). Moreover, the ROS generation sites overlap with mtDNA positions, which are mainly attached to the matrix side of the inner mitochondrial membrane (Murphy, 2012). This condition creates an opportunity to form mtDNA-protein crosslinks mediated by ROS, which increases mitochondrial fission and exacerbates mtDNA damage (Caston and Demple, 2017; Yang S.-G. et al., 2018). In turn, mtDNA damage and mutagenesis are directly responsible for a gradual impairment of the respiratory chain function and thus increase electron leakage and ROS production in the mitochondria. This “ROS vicious cycle” (Figure 2), present in different tissues and cells, causes exponentially accelerating oxidative stress with age (Pinto and Moraes, 2015). Consistent with this theory, Aitken et al. confirmed that in MII mouse oocytes, oxidative stress-catalyzed lipid peroxidation could initiate cyclic ROS transmission by directly destroying mitochondrial components (Lord et al., 2015). Studies also confirmed that exposure of mouse MII oocytes to exogenous H_2_O_2_ resulted in the dissipation of mitochondrial membrane potential, a decrease in cytoplasmic ATP levels, and the disruption of meiotic spindles (Zhang et al., 2006). However, H_2_O_2_-induced damage to mouse oocytes was ameliorated by supplementation with the antioxidant, NAC (Zhang et al., 2006). In addition, oxidative stress-induced cytochrome c (Cyt c) release from mitochondria, by changing the mitochondrial membrane potential, activates the apoptosis cascade (Redza-Dutordoir and Averill-Bates, 2016).

## Effects of Oxidative Stress on Inflammation in the Ovaries

### Inflammation and Ovarian Aging

Inflammation is associated with the pathogenesis of human aging (López-Otín et al., 2013; Franceschi et al., 2018b; Chung et al., 2019). This is also the case in the ovaries. Recent studies have shown that inflammation is a key marker of the aging ovarian stroma and is considered a new mechanism of POI (Huang et al., 2019b; Navarro-Pando et al., 2020). Clinical studies in humans have shown that inflammatory marker levels are associated with the risk of POI or early menopause. Ylmaz et al. detected the levels of serum inflammatory marker levels in patients with POI and normal fertile women, and found that the neutrophil-to-lymphocyte ratio (NLR) was significantly lower in the POI group than in the normal fertility group, and multivariate logistic regression analysis showed that NLR ≤ 1.5 was an independent risk factor for POI (Yldrm et al., 2015). Yue et al. also found that compared to the control group, the serum levels of interleukin (IL)-6 and IL-21 in the primary ovarian failure (POF) group were significantly higher (Sun et al., 2018). Chronic inflammation triggered by obesity might impair oocyte meiosis and oocyte quality (Snider and Wood, 2019). Consistent with these results, Whitcomb et al. found that early menopause cases had higher tumor necrosis factor receptor 2 (TNFR2) levels than controls (Bertone-Johnson et al., 2019). The concentration of TNFR2 was strongly related to TNFα and had the function of regulating TNFα activity (Yang S et al., 2018). Studies in animal models demonstrated that TNFα knockout mice had higher levels of GC proliferation, less incidence of follicle atresia, and higher fertility than wild-type mice (Cui et al., 2011). Therefore, inflammation also plays an important role in ovarian aging.

### Oxidative Stress and Inflammation in the Ovaries

**FIGURE 3:**
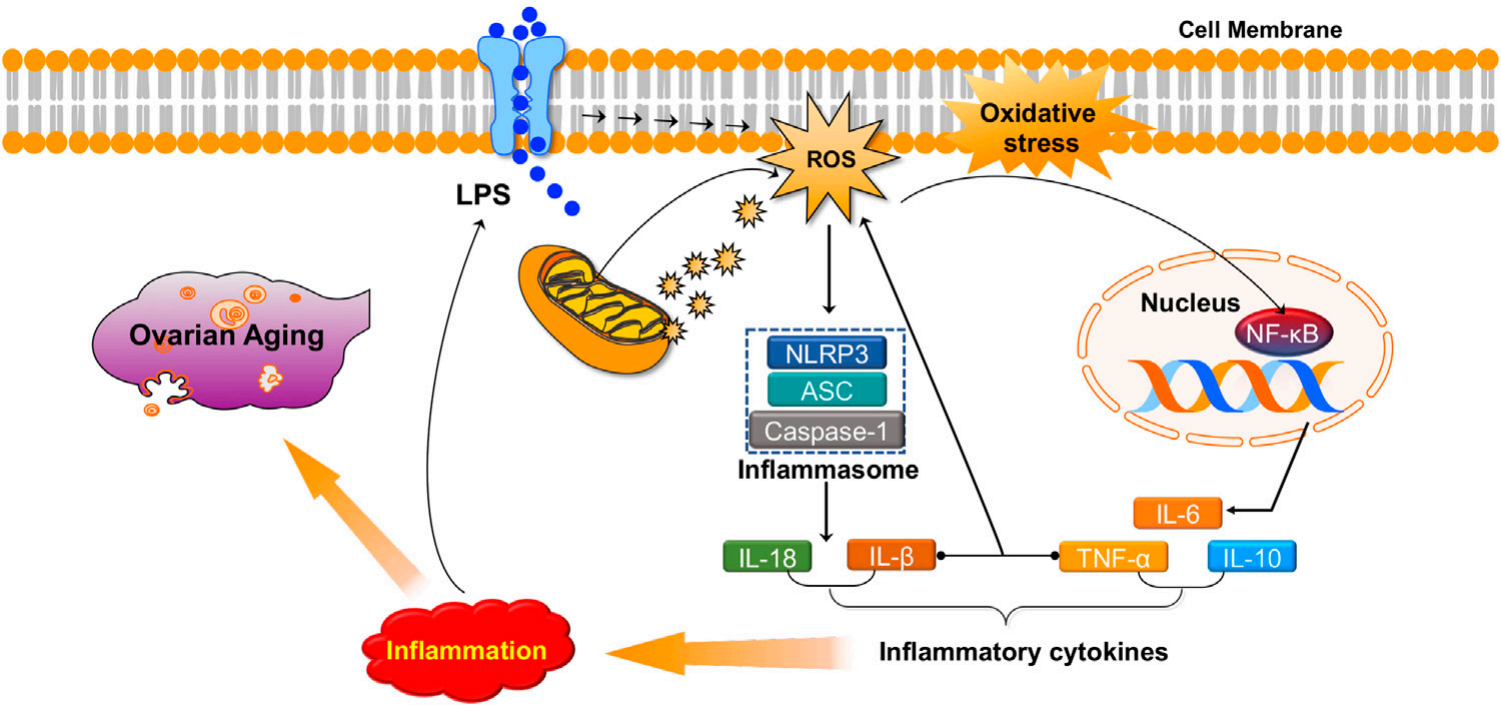
Oxidative stress promotes inflammation in the ovaries and causes ovarian aging.

Numerous studies have revealed that there is a strong association between inflammation and oxidative stress. They seem to accompany one another and promote each other in many chronic diseases (Li S et al., 2016; Biswas, 2016; Steven et al., 2019; Zuo et al., 2019). ROS may serve as a “kindling” to activate NLRP3 inflammasomes, leading to pro-inflammatory cytokine secretion (IL-1β and IL-18) (Abais et al., 2015; Long et al., 2020). ROS may also induce the activation of nuclear factor kappa B (NF-κB), a crucial mediator of inflammatory responses, and is associated with the pathogenesis of many disorders (Liu T et al., 2017; Liang et al., 2017; Qin et al., 2017). In addition, the inflammatory agents interferon (IFN)-γ and lipopolysaccharides (LPS) synergistically increase both extracellular and intracellular ROS production in human pancreatic cancer cells (Wu et al., 2013). Furthermore, studies have demonstrated that TNFα, IL-1β, and IFN-γ induce ROS production in multiple types of human cells (Yang et al., 2007; Agharazii et al., 2014). Similarly, increased levels of pro-inflammatory factors and oxidative products have been observed in animal POI models induced by chemotherapy drugs or radiotherapy, and antioxidats can simultaneously reduce pro-inflammatory factors (TNF α, IL-6, and IL-10) and oxidative product (malondialdehyde (MDA), H_2_O_2_, and ROS) levels by activating PI3K/AKT/mTOR or inhibiting NF-κB signaling pathways, thereby improving ovarian function (He et al., 2017; Liu C et al., 2018; Li and Liu, 2018; Jiang et al., 2019). In summary, oxidative stress can promote inflammation in the ovaries and cause ovarian aging (Figure 3).

## Effects of Oxidative Stress on Apoptosis in the Ovaries

### Apoptosis and Ovarian Aging

Apoptosis plays an important role in germ cell depletion in the mammalian ovaries (Tiwari et al., 2015). Follicular atresia caused by GC apoptosis is the primary process responsible for the loss of follicles and oocytes in the mammalian ovary and is one of the pathogenic mechanisms of premature ovarian failure (Krysko et al., 2008; Regan et al., 2018a; Regan et al., 2018b). Excessive GC apoptosis may deprive follicular oocytes of nutrients, maturation-enabling factors, and survival factors, and thus directly lead to follicular death (Regan et al., 2018a). Clinic studies have shown that higher mural GC apoptosis is correlated with decreased ovarian reserves, fewer oocytes retrieved, and a lower rate of high-quality embryos as well as with age (Sadraie et al., 2000; Fan et al., 2019). Consistent with this, Ebner et al. reported that the apoptotic GCs in younger patients (≤35 years) were significantly fewer than in older ones, and the processes of apoptosis seems to impair oocyte and gamete maturation (Corn et al., 2005). Animal experiments also provide strong evidence that the ovarian phenotype, including reduced ovulation rate and a dramatic decline in fertility, observed in hyh mutant (Napahyh/hyh) mice is based on an increased rate of apoptosis in GCs and follicular atresia (Arcos et al., 2017).

### Oxidative Stress and Apoptosis in the Ovaries

**FIGURE 4:**
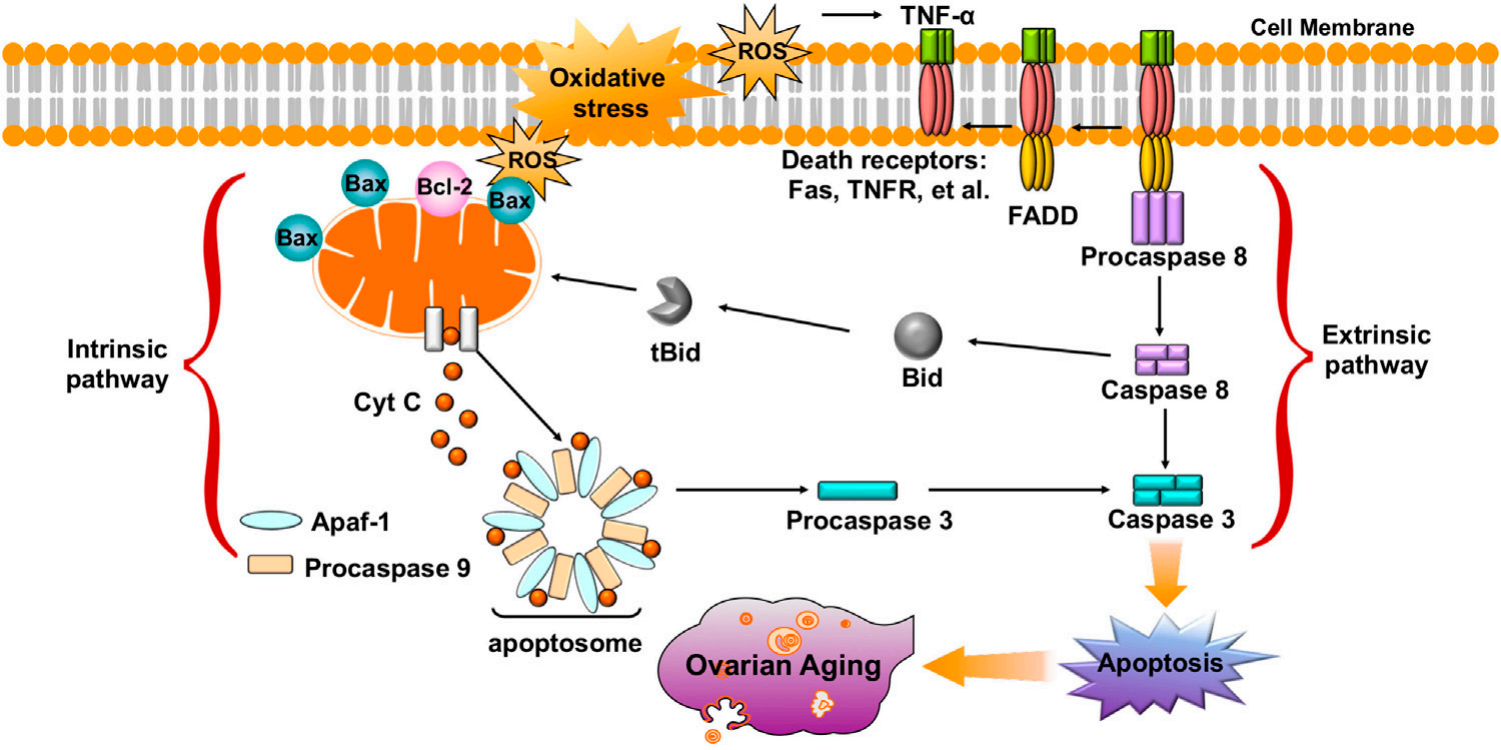
Oxidative stress leads to oocyte and GC apoptosis, and causes ovarian aging via extrinsic (death receptor) pathway and intrinsic (mitochondrial) pathway.

Accumulating evidence shows that oxidative stress is one of the key factors that induces oocyte and GC apoptosis in mammals (Prasad et al., 2016; Tiwari et al., 2016). To date, research indicates that there are two main pathways of oocyte and GC apoptosis caused by oxidative stress: the extrinsic (death receptor) pathway and the intrinsic (mitochondrial) pathway (Figure 4) (Regan et al., 2018a; Yadav et al., 2018). First, oxidative stress can induce the activation of mitochondrial pathways. Excessive ROS may change the mitochondrial membrane potential (MMP) by regulating the ratio of pro-/anti-apoptosis factors, which leads to the release of Cyt c from the mitochondria into the cytosol (Circu and Aw, 2010; Tripathi et al., 2013; Premkumar and Chaube, 2015). Cyt c further binds to apoptotic protease-activating factor 1 (APAF1), causing activation of caspase-9 (Monian and Jiang, 2012; Xiong et al., 2014). The effector, caspase-3, executes the final steps of apoptosis and cleaves various structural and regulatory proteins in female germ cells (Xiong et al., 2014). Li et al. demonstrated that oxidative stress induced bovine GC apoptosis by increasing the expression of cleaved caspase-3 and the Bax/Bcl-2 ratio as well as decreasing the expression of antioxidant enzymes (SOD2, glutathione peroxidase (GSH-Px)) (Wang Y et al., 2019). In addition, excessive ROS in oocytes and GCs can induce the release of TNFα (Kong et al., 2018; Miao et al., 2018). TNF-α binds to its death receptors (such as Fas and TNFR) and then activates the death receptor (extrinsic) pathway of apoptosis through Fas-associated death domain-dependent activation of caspase-8 (Morgan et al., 2008; Guicciardi and Gores, 2009; Redza-Dutordoir and Averill-Bates, 2016). Moreover, activated caspase-8 can also cleave Bid, which then causes the release of Cyt c from mitochondria and activates a crosstalk pathway between death receptors and mitochondria (Huang et al., 2016). Another study found that NAC counteracted H_2_O_2_-induced GC apoptosis via the ROS-JNK-p53 pathway, suggesting a functional role of ROS during this process (Yang et al., 2017). Therefore, protecting female germ cells against oxidative stress-induced apoptosis might be of great therapeutic value in the treatment of ovarian aging.

## Natural Antioxidant-Based Interventions to Reduce Oxidative Stress-Related Effects on the Ovaries

**FIGURE 5:**
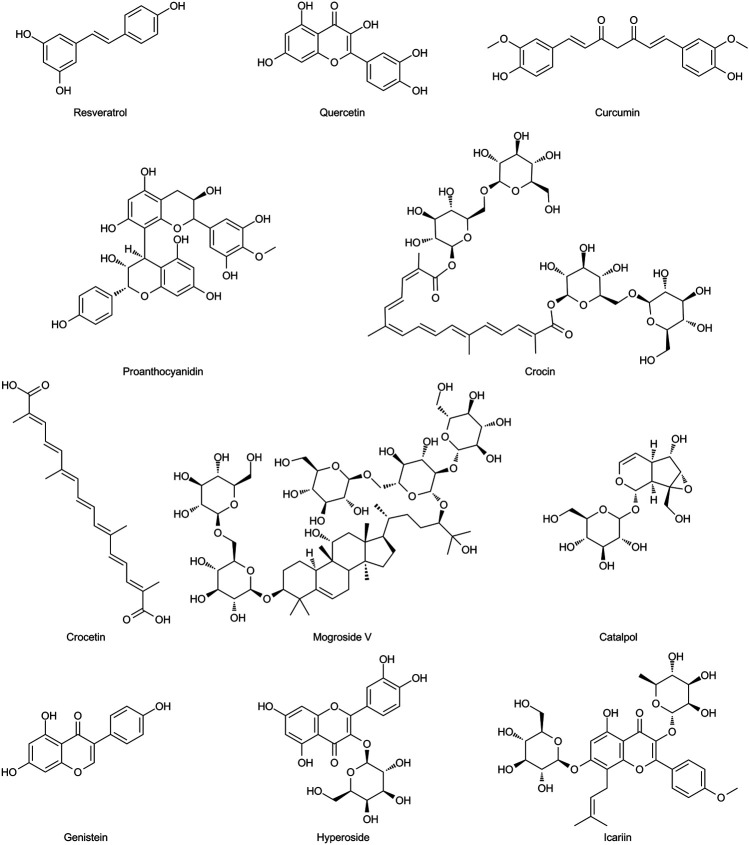
Chemical structures of the natural antioxidants summarized in this review.

Although aging has historically been viewed as an inevitable and unstoppable process, it is entirely possible to slow down the aging rate. Owing to the important role of oxidative stress in ovarian aging, an enormous amount of related research has focused on antioxidants. Medicinal plants have been used for thousands of years and represent an extraordinary inventory of high-diversity structural scaffolds in addition to being the most important source of antioxidants (Xu DP et al., 2017). Moreover, antioxidants from medicinal plants seem to act on various diseases, including ovarian aging, with their excellent anti-oxidative stress capacities. Some natural compounds with antioxidant properties that are isolated from medicinal plants have also been shown to protect against ovarian aging through multiple mechanisms (Xu X et al., 2017). A summary of these findings and the main efficacy indicators and mechanisms are summarized in Table 1 and Figure 5.

**TABLE 1:** Efficacy of natural antioxidants in protecting against ovarian aging.

Active ingredients	Experimental model	Efficacy	References
Resveratrol	MTX-induced ovarian-damaged rats	Counteracts MTX-induced cytotoxicity in rat ovary	Ata et al. (2019)
	Cisplatin-induced ovarian-damaged rats	Prevents the loss of the GCs	Said et al. (2019)
	Radiotherapy-induced POF rats	Restores ovarian function and diminishes ovarian inflammation	Said et al. (2016)
	Busulfan/cyclophosphamide-induced ovarian-aging mice	Improves ovarian aging and renewal capacity of oogonial stem cell	Wu et al. (2019)
	Rats exposed to chromium	Mitigates chromium-induced follicle atresia; rescues oocytes and GCs from apoptosis	Banu et al. (2016)
	VCD-induced diminished ovarian follicle rats	Improves VCD-induced DOR; increases the number of primary, primordial, and growing follicles	Ozatik et al. (2020)
	Mice exposed to Mancozeb	Alleviates Mancozeb induced infertility; increases ovary weight and primary follicles	Liu Y et al. (2017)
	C57/BL6 female mice under long-term oral administration of resveratrol	Increases primary and growing follicles; enhances oocyte quantity and quality in aged mice; counteracts age-related fertility decline	Liu et al. (2013)
	Aging oocytes of mice	Increases the rates of fertilization and blastocyst in POA oocytes; reduces the loss of sperm-binding sites; maintains the normal morphology of spindle and mitochondrion distribution; alleviates early apoptosis	Sun et al. (2019)
	Aged bovine oocytes and GCs	Improves the developmental progress of oocytes to the blastocyst stage; increases the mtDNA copy numbers and ATP content of oocytes	Sugiyama et al. (2015)
	Vitrified-warmed porcine oocytes	Improves survival, maturation, and MMP of vitrified-warmed oocytes	Ito et al. (2020)
Quercetin	CP-induced POF mice	Increases primordial follicles number and AMH level; decreases atretic follicles number	Elkady et al. (2019)
	Streptozotocin-induced diabetic mice	Increases the volume of the ovaries and growing follicles, significantly decreases the number of atretic follicles	Bolouki et al. (2019)
	Experimental ovarian ischemia-reperfusion injury rats	Prevents follicular cell degeneration, hemorrhage, vascular congestion, and edema	Gencer et al. (2014)
	Cadmium chloride-induced reproductive toxicity rats	Increases the number of follicles; decreases apoptosis of follicular cells	Nna et al. (2017)
	Goat oocytes	Improves mitochondrial activity and the percentage of MII oocytes; decreases the rate of apoptosis of MII oocytes	Silva et al. (2018)
	H_2_O_2_-induced oxidatively stressed human GCs	Significantly improves viability of human GCs; reduces the percentage of early apoptotic cells	Rashidi et al. (2019)
	H_2_O_2_-induced oxidatively stressed bovine GCs	Increases GC proliferation and mitochondrial activity; reduces intracellular ROS levels	Khadrawy et al. (2019)
	Oocytes of aging mice	Relieves deterioration in oocyte quality and improves subsequent embryo development	Wang H et al. (2017)
Curcumin	D-galactose-induced POF model mice	Increases the follicles at different developmental stages	Yan et al. (2018)
	Sodium arsenite-induced ovarian oxidative injury mice	Markedly reduces atretic follicles; increases the number of GCs	Wang XN et al. (2017)
	Exposed-to-whole-body ionizing radiation mice	Improves histological appearance of oocytes; reduces follicular atresia and GC apoptosis	Aktas et al. (2012)
	Zearalenone-induced oxidative stress porcine GCs	Rescues oxidative stress induced by Zearalenone	Qin et al. (2015)
	Estradiol valerate-induced PCOS rats	Significantly increases the number of primordial follicles, preantral, and corpus luteum; reduces the number of cysts and antral follicles	Mohammadi et al. (2017)
	CPM-induced POF rats	Significantly reduces atretic follicle, lipid peroxidation, hemorrhage around the corpus luteum, and vascular congestion in the ovarian stroma; improves histological parameters	Melekoglu et al. (2018)
	Rat ovaries with ischemia-reperfusion injury	Reverses tissue damage induced by ischemia-reperfusion injury in ovarian torsion	Sak et al. (2013)
Proanthocyanidin	D-gal-induced aging hens	Decreases the oxidative stress, alleviates the inhibition of aging on ovarian somatic cell proliferation, and decreases cell apoptosis	Liu X et al. (2018)
	3-NPA-induced ovarian oxidatively damaged mice	Significantly lower the percentage of GCs apoptosis and atretic follicles	Qin et al. (2015)
	Human GCs	Decreases oxidative stress and increases steroidogenesis	Barbe et al. (2019)
	Diquat-induced ovarian oxidatively damaged mice	Improves GCs viability, reduces GCs apoptosis rate, and induces autophagy process	Zhang et al. (2016)
Crocin	Mice oocytes	Improves IVM outcomes	Mokhber Maleki et al. (2014)
	Mouse oocytes	Improves nuclear maturation rates and subsequent developmental potential of mouse oocytes	Mokhber Maleki et al. (2014)
Crocetin	CPM-induced ovary injury mice	Reduces follicle loss; rescues fertility in CPM-treated mice	Di Emidio et al. (2017)
Mogroside V	Porcine oocytes	Improves IVM outcomes and subsequent embryonic development	Nie et al. (2020)
	Porcine oocytes	Protects porcine oocytes against *in vitro* aging	Nie et al. (2019)
Catalpol	Aged female rats	Nourishes ovarian tissues and improves both the quality and quantity of follicles	Wei et al. (2014)
	H_2_O_2_-induced oxidatively damaged rat ovarian GCs	Improves GCs viability	Yan et al. (2020)
Genistein	Pre-menopausal rats	Increases the number of surviving follicles and reserves; prolongs ovarian reproductive life	Zhuang et al. (2010)
	Ovarian toxicity rats induced by CPM	Decreases recruitment of primordial cells; increases the number of mature follicles and corpora lutea	Saleh and Mansour, (2016)
	ϒ-radiation-induced POF rats	Preserves all stages of healthy follicles; diminishes the atretic follicle population; enhances GC proliferation	Haddad et al. (2020)
Hyperoside	H_2_O_2_-stimulated Sprague-Dawley rats GCs	Improves GCs viability; protects GCs from H2O2-induced cell apoptosis and oxidative stress	Wang X et al. (2019)
Icariin	D-galactose-induced ovarian aging mice	Improves ovarian follicular development; inhibits follicular atresia; restores ovarian function of aging mice; enhances mice fertility	Wang J-L et al. (2019)

### Resveratrol

Resveratrol is a natural antioxidant polyphenol that is rich in the roots of *Reynoutria japonica* Houtt*.*, wine, and grapes (Meng et al., 2020). Resveratrol has received extensive attention in recent years owing to its wide range of biological properties, including anti-aging, anti-oxidation, anti-inflammatory, and anti-cancer activities (Wang et al., 2018; Tong et al., 2020; Yu et al., 2020). Growing evidence indicates that resveratrol has potential effects in stimulating ovarian function and retarding ovarian aging. In POF animal models, induced by drugs, radiotherapy, or heavy metals, resveratrol significantly promotes primordial follicle activation, increases the number of primary, primordial, and growing follicles, stimulates GC proliferation, diminishes ovarian inflammation, maintains ovarian architecture, and rescues oocyte and GC apoptosis (Banu et al., 2016; Said et al., 2016; Liu Y et al., 2017; Chinwe et al., 2018; Li and Liu, 2018; Ata et al., 2019; Said et al., 2019; Wu et al., 2019; Ozatik et al., 2020). Besides these features, long-term (12 months) oral administration of resveratrol can prevent telomere shortening, increase telomerase activity to increase the primary and growing follicles, increase the oocyte quantity and quality, and enhance embryonic development in aging mice to achieve anti-ovarian aging (Liu et al., 2013). Treatment with resveratrol during *in vitro* maturation (IVM) has been shown to reverse the adverse effects of oxidative stress on oocytes, improve blastocyst production from oocytes, increase mtDNA copy numbers, and elevate ATP content in oocytes and subsequent embryo development ability as well as raise the rates of fertilization in postovulatory aging (POA) oocytes, maintain the normal morphology of spindle and mitochondrion distribution, and alleviate early apoptosis (Itami et al., 2015; Sugiyama et al., 2015; Piras et al., 2019; Sun et al., 2019; Ito et al., 2020). These studies suggest that resveratrol might serve as an effective approach to improve ovarian functions and delay ovarian aging, and its specific effects and mechanisms are as follows: 1) increases nuclear factor erythroid 2-related factor 2 (Nrf2), a redox-sensitive transcription factor that regulates the expression of antioxidant genes (Espinosa-Diez et al., 2015), total glutathione (tGSH), SOD, and SOD2; reduces ROS production and MDA levels; and reverses the adverse effects of oxidative stress; 2) promotes mitochondrial synthesis, increases transcription factor a (TFAM), polymerase subunit gamma (POLG), and peroxisome proliferator-activated receptor gamma coactivator 1-α (PGC1α) levels; 3) regulates the mitochondrial apoptotic pathway, reduces the release of Cyt c and cleaved caspase-3, increases Bcl-2 and hypoxia-inducible factor 1-alpha (HIF1α), and inhibits oocytes and GC apoptosis; 4) acts as an anti-inflammation agent by inhibiting NF-κB-provoked inflammatory cytokines (IL-6, IL-8); 5) prevents telomere shortening and increases telomerase activity; and 6) regulates the expression of the SIRT1 gene while activating the PI3K/Akt signaling pathway.

### Quercetin

Quercetin, a bioactive flavonoid, is widely found in medicinal plants and foods, such as *Ginkgo biloba* L*.*, *Hypericum perforatum* L*.*, apples, berries (Li Y et al., 2016). It has extensive biological properties, including antioxidant, anti-inflammatory, and anti-apoptosis effects, and stimulates mitochondrial biogenesis (Li Y et al., 2016; Xu et al., 2019; Zeng et al., 2020). Recently, the use of quercetin in anti-ovarian aging treatment has been increasingly considered.

In laboratory animals, quercetin has been shown to increase the volume of the ovary and the primordial follicle number, the number of growing follicles along with the corpus luteum, and prevent follicular cell degeneration, hemorrhage, vascular congestion, and edema while decreasing apoptosis of follicular cells (Gencer et al., 2014; Nna et al., 2017; Bolouki et al., 2019; Elkady et al., 2019). In addition, in multiple *in vitro* studies using animal and human GCs, quercetin treatment enhanced viability of GCs, reduced the percentage of early apoptotic cells, relieved deterioration in oocyte quality, and improved subsequent embryo development (Wang H et al., 2017; Silva et al., 2018; Khadrawy et al., 2019; Rashidi et al., 2019). Accordingly, quercetin has a protective role in the ovaries,and its specific effects and mechanisms are as follows: 1) enhances Nrf2, SOD1, CAT, glutathione synthetase (GSS), GSH, and glutathione peroxidase (GPx) activity; reduces ROS production and MDA levels; and inhibits oxidative stress; 2) decreases anti-apoptotic caspase levels; 3) inhibits the TLR/NF-κB inflammation signaling pathway; and 4) enhances mitochondrial activity.

### Curcumin

Curcumin is the main natural polyphenol extracted from the rhizome of *Curcuma longa* L., which has been traditionally used in Asian countries as a medical herb for thousands of years (Kotha and Luthria, 2019). Curcumin is a widely studied nutraceutical and is known recently to have antioxidant, anti-aging, anti-inflammatory, anti-apoptosis, and anti-cancer functions (Kocaadam and Şanlier, 2017; Bielak-Zmijewska et al., 2019; Boarescu et al., 2019). In laboratory animals, curcumin has been shown to play a stimulatory role in ovarian functions and prevents the compromise of ovarian functions caused by cyclophosphamide (CPM), ionizing radiation, and ischemia (Alekseyeva et al., 2011; Aktas et al., 2012; Sak et al., 2013; Qin et al., 2015; Wang XN et al., 2017; Mohammadi et al., 2017; Melekoglu et al., 2018; Yan et al., 2018). Treatment with curcumin has been shown to increase the number of follicles at different developmental stages and in GCs, improve histological appearance of oocytes, and markedly reduce atretic follicles, lipid peroxidation, hemorrhage around the corpus luteum, and vascular congestion in the ovarian stroma (Alekseyeva et al., 2011; Aktas et al., 2012; Sak et al., 2013; Qin et al., 2015; Wang XN et al., 2017; Mohammadi et al., 2017; Melekoglu et al., 2018; Yan et al., 2018). The specific effects and mechanisms involved are as follows: 1) alleviates ovarian oxidative injury, increases the levels of Nrf2, heme oxygenase-1(HO-1), SOD, and SOD1 while reducing ROS production and MDA levels; 2) decreases anti-apoptotic levels of caspase-3 and -9; and 3) as an anti-inflammation agent, reduces the levels of TNF-α, IL-6, and CRP.

### Proanthocyanidin

Proanthocyanidin (PA) is an important class of polyphenols abundant in grape seeds, *Lycium ruthenicum* Murry., and blueberries, possessing antioxidant, anti-inflammatory, and anti-cancer activities (Shi et al., 2003; He et al., 2018). In hens, PA treatment significantly alleviated the inhibition of ovarian somatic cell proliferation and decreased cell apoptosis in D-gal-induced and natural aging ovarian tissues by reducing oxidative stress (Liu X et al., 2018). Similarly, in 3-nitropropionic acid (3-NPA)-induced oxidative ovarian damaged mice, PA significantly reduced the percentage of GC apoptosis and atretic follicles in ovarian tissues, increased the expression of antioxidant genes, and inhibited the expression of pro-apoptotic genes (Zhang JQ et al., 2015). In addition, in multiple *in vitro* studies using animal and human GCs, PA and procyanidin B2 (B type of PA) exert a potent effect in terms of diminishing GC apoptosis and intracellular ROS production (Zhang et al., 2016; Barbe et al., 2019). Taken together, the administration of PA has the potential to alleviate ovarian oxidative injury and delay ovarian aging mainly through antioxidant and anti-apoptotic activities.

### Crocin and Crocetin

Crocin and crocetin are both carotenoid chemical compounds of *Crocus sativus* L. and are well-known in traditional medicine (Leone et al., 2018). They show high radical-scavenging activity as well as anti-inflammatory, anti-apoptosis, and probably anti-aging activity (Assimopoulou et al., 2005; Bukhari et al., 2018). Abedi et al. demonstrated that supplementation of IVM media with crocin significantly reduces the adverse effects of oxidative stress, thereby improving nuclear maturation rates and subsequent developmental potential of mouse oocytes (Mokhber Maleki et al., 2014; Mokhber Maleki et al., 2016). In addition, crocetin administration reduces follicle loss and rescues fertility in CPM-treated mice. Mechanistically, crocetin protects the ovary against CPM by modulating redox balance, decreasing SIRT3, and increasing the antioxidant enzyme, SOD2, as well as the mitochondrial biogenesis activator, PGC1α (Di Emidio et al., 2017).

### Mogroside V

Mogroside V (MV) is the most abundant form of triterpenoid compound isolated from *Siraitia grosvenorii* (Swingle) C.Jeffrey ex A.M.Lu & Zhi Y.Zhang (Liu H et al., 2018; Gong et al., 2019). Numerous studies have demonstrated that MV possesses broad pharmacological characteristics and properties, including antioxidant, anti-tussive, immunoregulatory, and anti-inflammatory effects (Di et al., 2011; Itkin et al., 2016; Liu C et al., 2018). Liang et al. reported that supplementation of IVM media with MV significantly increased the IVM rate and subsequent embryonic development. Furthermore, MV reduced intracellular ROS and increased the mRNA expression of oxidative stress-related genes (SOD, CAT, and SIRT1) while enhancing mitochondrial function (Nie et al., 2020). MV markedly reduces the decline in porcine oocyte quality during *in vitro* aging, possibly by reducing oxidative stress and early apoptosis in aged oocytes while improving mitochondrial contents and function (Nie et al., 2019).

### Catalpol

Catalpol is an iridoid glycoside abundant in the roots of *Rehmannia glutinosa* (Gaertn.) DC. and has been shown to possess a broad range of bioactivities, especially antioxidative effects, and is considered a potential candidate for treating oxidative stress-induced neurodegenerative disease (Jiang et al., 2015; Tong et al., 2015; Zheng et al., 2017). Recently, the protective role of catalpol in the ovaries has also been confirmed. Studies have shown that catalpol can nourish ovarian tissue and improve the quality and quantity of follicles and has a direct anti-aging effect on the rat ovarian system (Wei et al., 2014). Catalpol also improved GC viability and protected GCs from H_2_O_2_-induced oxidative injury and apoptosis (Yan et al., 2020). The mechanism likely involved is that catalpol inhibits H_2_O_2_-induced ROS, MDA, Bax, and caspase-9 production while activating SOD, GSH-Px, and Bcl-2 expression (Yan et al., 2020).

### Genistein

Genistein, one of the most important isoflavones, is found in soybean products, and in the traditional medicinal plants, including *Pueraria montana* (Lour.) Merr., *Cistanche deserticola* Ma, and *Eucommia ulmoides* Oliv. (Park et al., 2019; Tang et al., 2019). It shows estrogenic properties via binding to different estrogen receptors and has various biological effects in antioxidation and anti-aging (Spagnuolo et al., 2015; Liu et al., 2020). Indeed, *in vitro* studies have shown that genistein increased the number of follicles surviving as reserves and prolonged ovarian reproductive life (Zhuang et al., 2010). In addition, genistein alleviated the oxidative stress and inflammation in CMP-induced ovarian toxicity rats (Saleh and Mansour, 2016). Specifically, genistein decreased the recruitment of primordial cells and increased the number of mature follicles and corpora lutea by upregulating ovarian SOD and GSH levels, and downregulating IL-1β and MAD levels, which means preservation of ovarian function and follicular reservoirs (Saleh and Mansour, 2016). Moreover, compared with estradiol, genistein was superior in preserving all stages of healthy follicles, enhancing GCs proliferation, and reducing the population of atretic follicles in ϒ-radiation-induced POF rats (Haddad et al., 2020).

**FIGURE 6:**
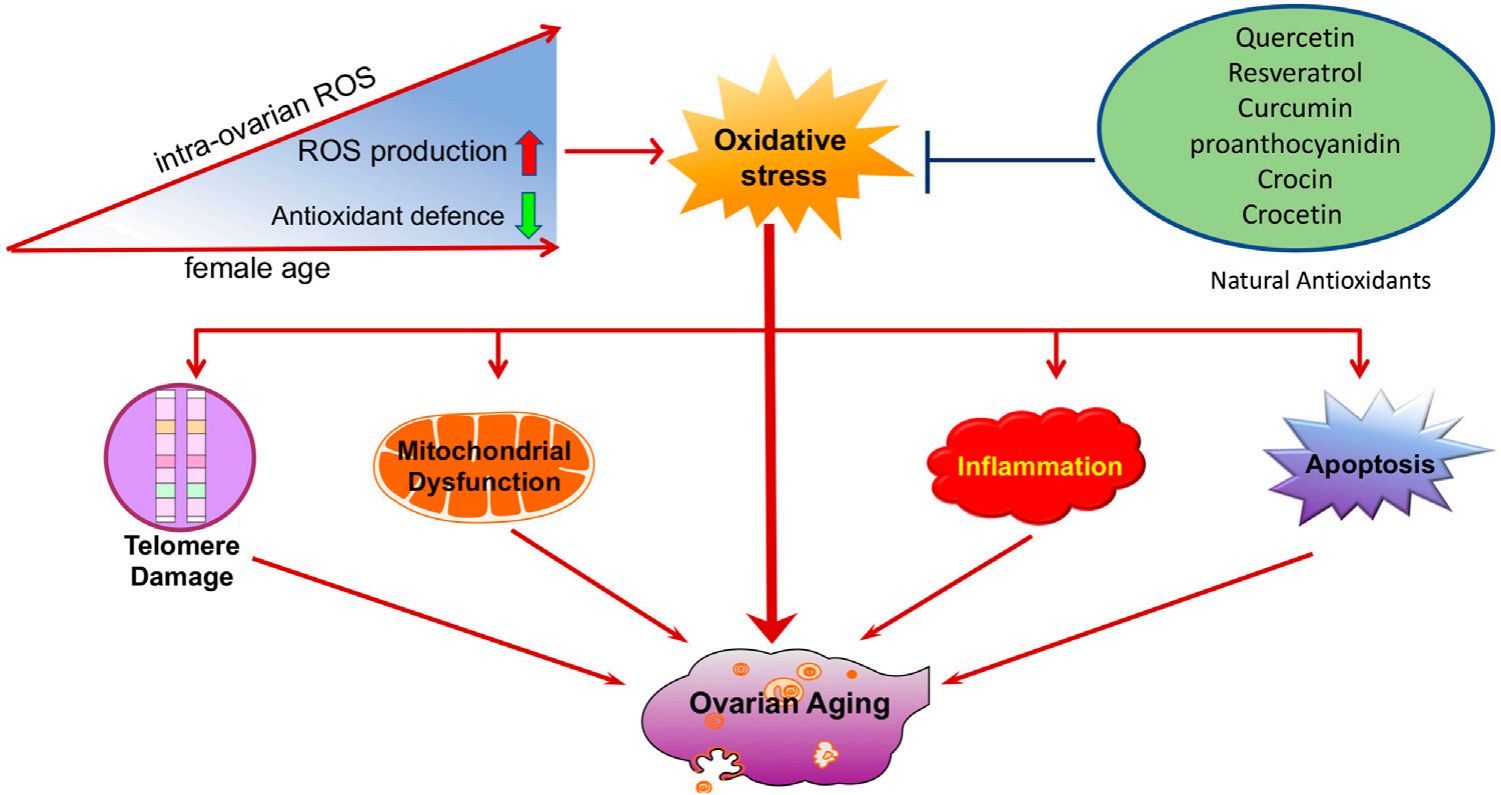
Schematic illustration of oxidative stress during ovarian aging. Oxidative stress promotes the development of other ovarian aging-related etiologies, including telomere shortening, mitochondrial dysfunction, apoptosis, and inflammation. Natural antioxidants supplements, such as resveratrol, quercetin, and curcumin might be beneficial in preventing ovarian aging.

There are several other natural compounds with antioxidant, anti-inflammatory, anti-apoptosis, and anti-aging properties, such as hyperoside and icariin, which may also exert beneficial effects on ovarian aging (Wang J-L et al., 2019; Wang X et al., 2019). The chemical structures of the natural antioxidants summarized in this review are presented in Figure 6.

Apart from natural compounds, various diets rich in antioxidants are also sources of natural antioxidants. For example, kiwifruit, nuts, tomatoes are rich in vitamins C, D, and E; walnuts, peanuts, cherries, corn are high in melatonin. A prospective study showed that there is a highly significant correlation between vitamin D deficiency and DOR (Arefi et al., 2018). The combined use of vitamin E and C can prevent apoptosis of ovarian tissue following mancozeb exposure in the first generation of mouse pups (Mahdi et al., 2019). In addition, melatonin may act as a free radical scavenger and has antioxidant properties. Its role in anti-ovarian aging has been widely reported. Oral melatonin can ameliorate intrafollicular oxidative stress, improve the quantity and quality of oocytes and IVF outcomes (Jahromi et al., 2017; Espino et al., 2019; Hu et al., 2020). In contrast, night shift work may lead to impaired pineal function and inhibit melatonin production (James et al., 2017). Teixeira et al. reported that night shift workers have a higher level of oxidative stress damage and a lower level of antioxidant defenses (Teixeira et al., 2019). Women who rotate night shift work have an increased risk of early menopause and ovarian failure (Stock et al., 2019). Therefore, healthy sleep also contributes to antioxidant stress and anti-ovarian aging.

## Conclusion

Driven by societal trends, many young women choose to postpone marriage and/or childbirth. Age-related fertility issues have become serious challenges in reproductive medicine because aging causes a reduction in both oocyte quality and quantity. Oxidative stress is a crucial factor in ovarian functional decline with age. It acts as a driver of the etiology of ovarian aging. Compelling evidence has shown that oxidative stress promotes the development of other ovarian aging-related etiologies, including telomere shortening, mitochondrial dysfunction, apoptosis, and inflammation, which provides new insights into our understanding of the mechanisms of ovarian aging.

**FIGURE 7:**
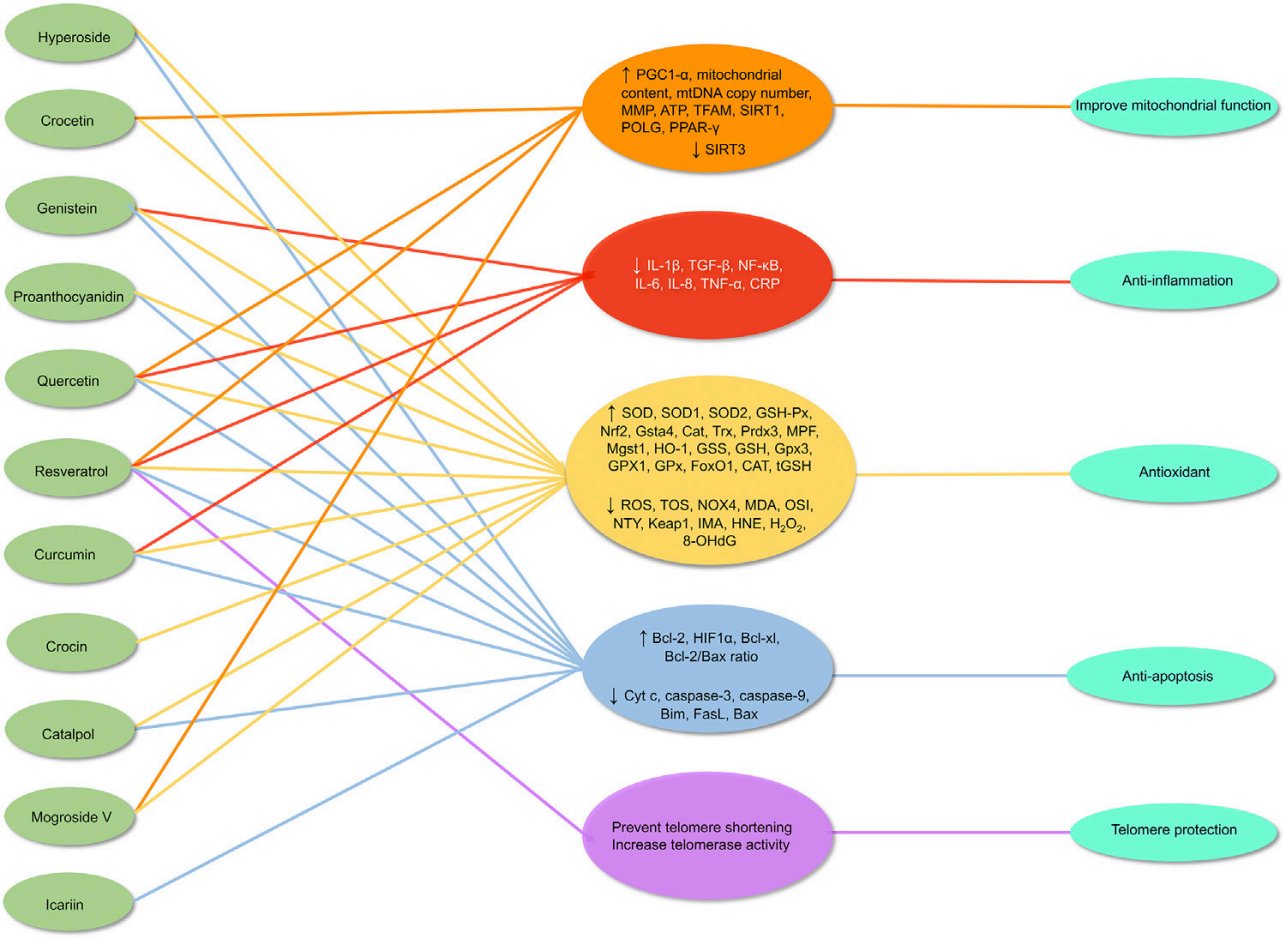
Mechanism of natural antioxidants in protecting against ovarian aging through multiple mechanisms. ↑ = up-regulates, ↓ = down-regulates.

Therefore, alleviating oxidative stress in the ovaries is an important entry point for delaying ovarian aging. Compared with synthetic antioxidants, antioxidants from natural products have a high anti-oxidative stress capacity and are safe and acceptable. In our review, certain natural antioxidants have been tested *in vivo* and *in vitro* with promising results and multiple mechanisms. These findings raise the prospect of oxidative stress modulatory natural antioxidants as therapeutic interventions for delaying ovarian aging (Figure 7). While substantial research supports these strategies, further investigation is warranted, particularly through clinical trials.

## Author Contributions

QZ conceptualized the manuscript. LY, XC, YC,YL, LY, YX, CM, and YZ collected the literature, wrote the manuscript, and prepared figures. QZ edited and made significant revisions to the manuscript. All authors contributed to the article and approved the submitted version.

## Funding

QZ got the Programs: Zhejiang Provincial TCM Sci-tech Plan (2020ZA078) and Zhejiang Zhangqin famous Traditional Chinese Medicine expert inheritance studio project (GZS2012014). YC got the Program: Medical and health science and technology plan of Zhejiang Province (2021KY920).

## Conflict of Interest

The authors declare that the research was conducted in the absence of any commercial or financial relationships that could be construed as a potential conflict of interest.

## Glossary

ART: assisted reproductive technology
ATP: adenosine triphosphate
CAT: catalase
CPM: cyclophosphamide
Cyt c: cytochrome c
DOR: diminished ovarian reserve
FADD: fas-associated death domain
GCs: granulosa cells
GPx: glutathione peroxidase
GPx3: glutathione peroxidase 3
GSH: glutathione
GSH-Px: glutathione peroxidase
GSS: glutathione synthetase
Gsta4: glutathione transferase α4
H2O2: hydrogen peroxide
HIF1α: hypoxia-inducible factor 1-alpha
HO-1: heme oxygenase-1
IFN-γ: interferon gamma
IL-18: interleukin 18
IL-1β: interleukin 1 beta
iNOS: inducible nitric oxide synthase
IVM: in vitro maturation
LPS: lipopolysaccharides
MDA: malondialdehyde
Mgst1: microsomal glutathione S-transferase 1
MMP: mitochondrial membrane potential
MPF: maturation promoting factor
MTX: methotrexate
MV: Mogroside V
NAC: N-acetylcysteine
NF-κB: nuclear transcription factor B
3-NPA: 3-nitropropionic acid
Nrf2: nuclear factor erythroid 2-related factor 2
PA: Proanthocyanidin
PCOS: polycystic ovary syndrome
PGC1α: peroxisome proliferator-activated receptor gamma coactivator 1-α
POA: postovulatory aging
POF: primary ovarian failure
POI: primary ovarian insufficiency
POLG: polymerase subunit gamma
Prdx3: peroxiredoxin 3
ROS: reactive oxygen species
SIRT1: nicotinamide adenosine dinucleotide-dependent deacetylase sirtuin-1
SODs: auperoxide dismutases
TAS: total antioxidant status
TFAM: transcription factor a
tGSH: total glutathione
TNF α: tumor necrosis factor-alpha
TNFR2: tumor necrosis factor receptor 2
TOS: total oxidant status
